# Crossing Frequency Method Applicable to Intermediate Pressure Plasma Diagnostics Using the Cutoff Probe

**DOI:** 10.3390/s22031291

**Published:** 2022-02-08

**Authors:** Si-jun Kim, Jang-jae Lee, Young-seok Lee, Chul-hee Cho, Shin-jae You

**Affiliations:** 1 Nanotech Optoelectronics Research Center, Yongin 16882, Korea; kim_sijun@naver.com; 2 Applied Physics Lab for PLasma Engineering (APPLE), Department of Physics, Chungnam National University, Daejeon 34134, Korea; leejj3800@naver.com (J.-j.L.); ys.dunphy@gmail.com (Y.-s.L.); paulati@naver.com (C.-h.C.); 3 Institute of Quantum Systems (IQS), Chungnam National University, Daejeon 34134, Korea

**Keywords:** plasma diagnostics, electron density measurement, cutoff probe, high-pressure plasma, crossing frequency method

## Abstract

Although the recently developed cutoff probe is a promising tool to precisely infer plasma electron density by measuring the cutoff frequency ($f_{\mathrm{cutoff}}$) in the S${}_{21}$ spectrum, it is currently only applicable to low-pressure plasma diagnostics below several torr. To improve the cutoff probe, this paper proposes a novel method to measure the crossing frequency ($f_{\mathrm{cross}}$), which is applicable to high-pressure plasma diagnostics where the conventional $f_{\mathrm{cutoff}}$ method does not operate. Here, $f_{\mathrm{cross}}$ is the frequency where the S${}_{21}$ spectra in vacuum and plasma conditions cross each other. This paper demonstrates the $f_{\mathrm{cross}}$ method through three-dimensional electromagnetic wave simulation as well as experiments in a capacitively coupled plasma source. Results demonstrate that the method operates well at high pressure (several tens of torr) as well as low pressure. In addition, through circuit model analysis, a method to estimate electron density from $f_{\mathrm{cross}}$ is discussed. It is believed that the proposed method expands the operating range of the cutoff probe and thus contributes to its further development.

## 1. Introduction

Composed of physically energetic charged particles and chemically reactive neutral particles, plasma has been widely used in various fields including material fabrication and nuclear fusion as well as medical, environmental, and aerospace industries [1,2]. Plasma processing techniques such as plasma etching [3,4,5,6,7], ashing [8,9,10,11], and deposition [12,13,14,15,16] are the most important steps to fabricate the high-end memory and system semiconductors used in internet of things and artificial intelligence technologies. For plasma deposition in particular, plasma sputtering, plasma-enhanced chemical vapor deposition (PECVD), and plasma-enhanced atomic layer deposition (PEALD) approaches have been widely used for their high deposition rates, low-temperature processing, good film conformality, and high film uniformity [12,13,17,18].

In conventional deposition processing, trial-and-error methods were first adopted to find the optimum process window [19]. However, these days, such an approach seems ill-suited since the current challenges in cutting-edge material fabrication involve processing steps that abruptly increase and also involve complicated chemistries [1,3,4,12,13]. To overcome this limitation, two alternatives have been proposed, namely computer simulation and plasma internal parameter diagnostic methods [20].

For the former, accompanied by the explosive improvements in computing power, multiphysics methods allow us to simulate the plasma deposition process, predict the processing results from plasma sputtering [21], PECVD [22], and PEALD [23], and finally estimate the optimum process window based on the results. However, there remains a lack of basic atomic data especially for new complex precursors such as bulk reaction cross sections, surface coefficients, and sputtering and secondary electron emission yields [24]. Consequently, simulation is at present applicable to specific processes using simple chemistries.

The latter, referring to methods that find the optimum process window based on internal plasma parameters, has attracted great interest in industrial as well as academic fields since plasma has an influence on most chemical reactions contributing to the deposition process [25,26,27,28]. Specifically, electrons produce chemical species, which play a dominant role in deposition chemistry, while energetic ions and metastables activate the material surface, which enhances surface chemical reactions [12,19]. Here, the plasma internal parameters include electron density, electron temperature, ion flux, and ion energy distribution. Among them, electron density is known as one of the most important parameters because it is directly related to the deposition rate and processing productivity [29,30].

Various plasma diagnostics to measure electron density have been developed, such as the Langmuir probe measuring electron and ion current [31,32], the line ratio method analyzing optical emission spectra by excited atoms and molecules [33], the laser Thomson scattering method measuring scattered laser light by electrons [34], and microwave probes analyzing absorbed, reflected, and transmitted microwave signals [35,36,37,38]. The Langmuir probe can infer various electron characteristics such as electron density, temperature, and energy distribution, but is not applicable to deposition processing since films deposited on the probe tip block the conduction current, and the design of an RF filter to block high-frequency noise is difficult [39]. As for the optical methods, while the measurement of a plasma emission spectrum through a viewport in a process chamber is relatively simple, these approaches are only applicable to a narrow processing window since the emission spectrum by each species overlaps, which complicates spectra analysis [33]. The laser Thomson scattering method requires a large and stable space to generate the laser and detect scattered light since the detection signal is small. Furthermore, both optical and laser methods are vulnerable to deposition on the viewport, which induces a decrease in emission and scattered light signals.

On the other hand, the microwave probes are free from the issue of film deposition on the probe antenna [40] as displacement current can flow through a dielectric film. Furthermore, the microwave signal only slightly distorts the processing plasma because the probes operate at relatively low power, i.e., <1 mW [41], in comparison to common RF powers ranging from several hundreds to thousands of watts. Hence, microwave probes are seen as useful tools in deposition processing, and various types of probes have been developed. Details of these probes, such as the curling probe (CLP), the multipole resonance probe (MRP), the cutoff probe (CP), etc., are well explained in [42,43].

Recently, microwave probes have been applied to measure the electron density of deposition plasma. Styrnoll et al. applied the MRP to the ion-assisted deposition used in optical coatings at a relatively low pressure (<0.2 Torr) [44]. Ogawa et al. applied the CLP to a hydrogenated amorphous carbon film deposition process (<0.01 Torr) [45], and Lee et al. applied the CP to a fluorocarbon film deposition process (<0.02 Torr) [7]. The probes in these works showed good performance in measuring electron density during the deposition process at low pressure.

However, based on the analysis in [46], the CLP and MRP might be seen as unsuitable for high-pressure applications. The CP, likewise, has a limitation for high-pressure plasma measurement [47,48,49]. Probe performance reduction in such an environment results from frequent electron–neutral collisions that decrease plasma–electromagnetic wave interaction and diminish the resonant character of the probe system. Accordingly, there is a great demand for the means to measure high-pressure deposition plasma to analyze and optimize the deposition process.

Considering that the CP is a promising microwave probe showing high reproducibility and high accuracy [43,50,51], improvements of the CP for high-pressure plasma measurement are highly desirable. In the current paper, an alternative method to measure electron density using the CP in a high-pressure condition is proposed, called the crossing frequency method.

The remainder of this paper is as follows. In the second section, simulation analysis for the crossing frequency method is given. In the third section, experimental validation of the proposed method is given, and a simple relation between electron density and crossing frequency is presented. Finally, in the fourth section, a summary of this paper is provided.

## 2. Simulation Demonstration

A commercial software to solve Maxwell’s equations in three-dimensional space, Computer Simulation Technology (CST) MicroWave Studio Suite, was adopted in this study. CST simulation is based on a finite-difference time-domain method and is quite accurate compared with experiments [50]. This simulation considers plasma as a dispersive dielectric material, called the Drude model, in which ions and electrons are immobile and freely mobile, respectively, with a plasma dielectric constant ($\epsilon_{p}$) given by

(1) $$\epsilon_{p}\left(\omega \right)=\epsilon_{0}\left(1-\frac{2\pi f_{\mathrm{pe}}}{\omega \left(\omega -j\nu_{m}\right)}\right),$$

where *j* is a complex number, $\epsilon_{0}$ is the vacuum dielectric constant, ω is the microwave frequency, $f_{\mathrm{pe}}$ (=8980$\sqrt{n_{e}}$) is the plasma oscillation frequency, $n_{e}$ is the electron density in units of cm${}^{-3}$, and $\nu_{m}$ is the electron–neutral collision frequency. Here, the dispersive dielecrtic material means that its dielectric constant has frequency dependence. Equation (1) is used to solve Maxwell’s equation inside the plasma domain. Here, for simplicity, it is assumed that the electron–neutral elastic collision is only considered for the calculation of $\nu_{m}$ between an electron and argon atom at an electron temperature of 2 eV and a gas temperature of 300 K. The 2 eV is a mean value commonly used in plasma processing. A Maxwellian electron energy distribution is also assumed. Then, $\nu_{m}$ is equal to 2.26×p in MHz, where *p* is the pressure in mTorr.

CST simulation provides several boundary conditions including electrical ground, magnetic ground, open, and periodic conditions. To study microwave probes, the electrical ground and open boundary conditions are the most commonly used. To include vacuum chamber effects, the former is usually applied, while to only consider the principal effect without any boundary effects, the latter is typically used. In the current work, the purpose of the simulation-based study was to investigate the basic operation of the crossing frequency method, and so the open boundary condition was applied to all simulation domain boundaries.

**Figure 1:**
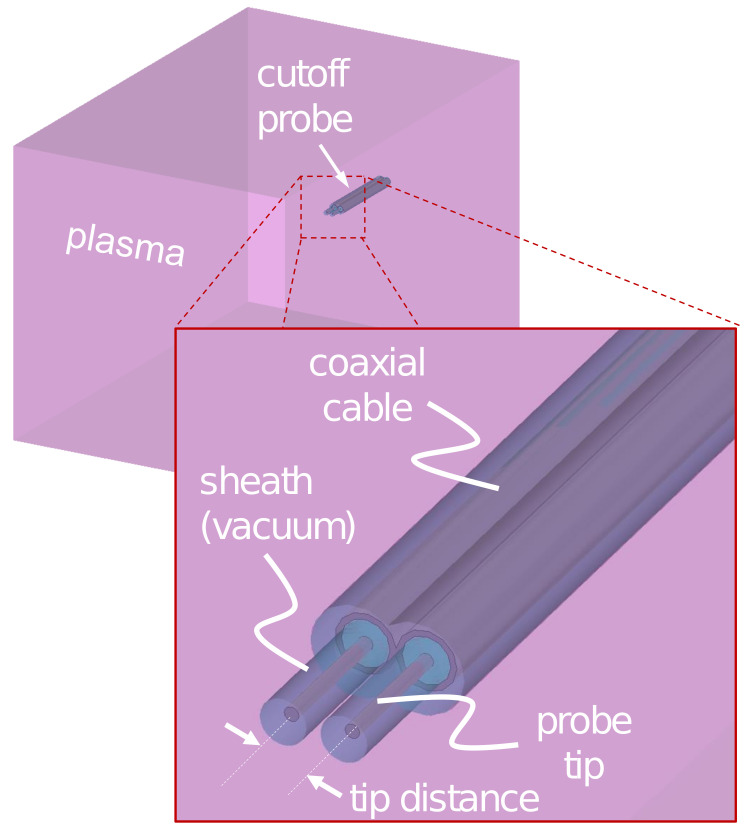
Configuration in a three-dimensional electromagnetic wave simulation.

Figure 1 shows the simulation configuration, where the CP is immersed in a uniform rectangular plasma (100 × 100 × 150 mm${}^{3}$) having an $n_{e}$ of $5\times 10^{10}$ cm${}^{-3}$. The CP consists of radiating and detecting tips that are each connected to separate coaxial cables, which play the role of a transmission line with characteristic impedance of 50 Ω. The plasma dimension is larger than the skin depth of the microwaves ranging from 0 to 5 GHz, so the plasma shape is not an important factor. The probe tip length, distance, and radius are 5.0 mm, 4.0 mm, and 0.26 mm, respectively, and the length of the coaxial cables is 30.0 mm. The sheath covering the cutoff probe is considered as a vacuum with a dielectric constant of $\epsilon_{0}$ and a width of 0.234 mm, which is the same as the floating sheath width, 5 $\times \;\lambda_{\mathrm{De}}$, where $\lambda_{\mathrm{De}}$ is the Debye length [52].

The S${}_{21}$ spectrum calculation in this simulation is as follows. A Gaussian pulse signal including microwaves from 0 to 5 GHz enters the end of the coaxial cable and proceeds toward the radiation tip with radiation power P${}_{\mathrm{rad}}\left(t\right)$. The pulse signals are reflected at the plasma–sheath interface, absorbed inside the plasma, and transmitted through the plasma by way of evanescent waves to the detection tip with detection power P${}_{\det}\left(t\right)$. Then, each power in the time domain is transformed via the fast Fourier transform method in the frequency domain as p${}_{\mathrm{rad}}\left(f\right)$ and p${}_{\det}\left(f\right)$. The S${}_{21}$ spectrum can then be calculated by 10log${}_{10}\left(p_{\det}\left(f\right)/p_{\mathrm{rad}}\left(f\right)\right)$.

**Figure 2:**
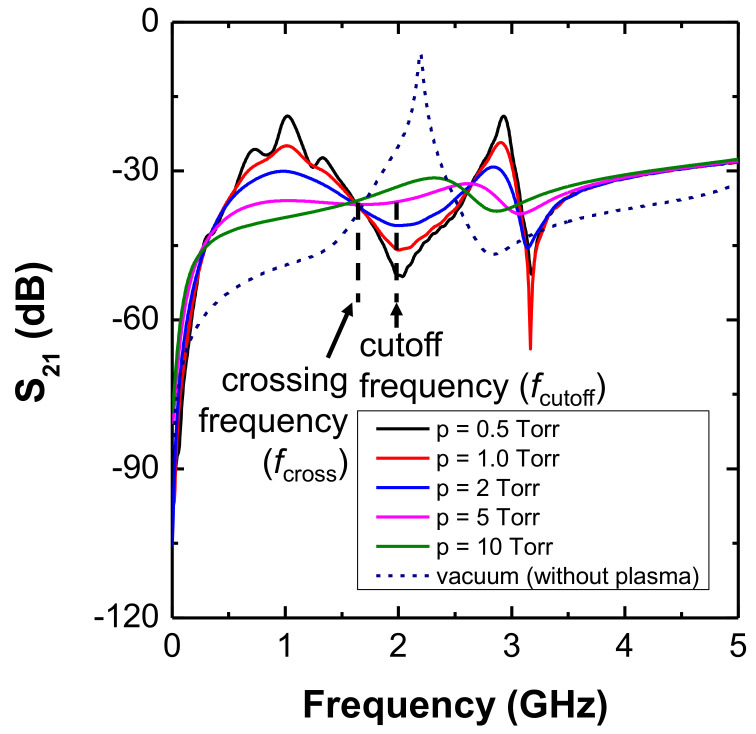
Simulated S${}_{21}$ spectra at various pressures with an electron density of 5 × 10${}^{10}$ cm${}^{-3}$, tip length of 5.0 mm, sheath width of 0.234 mm, and tip distance of 4 mm.

Figure 2 shows S${}_{21}$ spectra at vacuum and various pressure conditions. Here, the vacuum condition means that there is no plasma and the CP is immersed in a vacuum material whose dielectric constant is $\epsilon_{0}$. There is a clear resonance peak (maximum S${}_{21}$ value) in the vacuum spectrum near 2 GHz that results from the quarter-wavelength resonance by the coaxial cable, which is the same as in [53]. The simple estimation that the length of the coaxial cable is 30 mm and the quarter-wavelength of 2 GHz is about 30 mm support this quarter-wavelength resonance. Otherwise, in the case of plasma (<10 Torr), there is a clear cutoff peak in the S${}_{21}$ spectrum as marked in Figure 2, and this frequency is defined as the cutoff frequency, $f_{\mathrm{cutoff}}$. As the pressure increases, the cutoff peak broadens before finally disappearing above 5 Torr [47], which is known as the pressure limitation of the CP. That is, the conventional method measuring $f_{\mathrm{cutoff}}$ can operate below several torr.

We note here that there is a specific frequency where the vacuum spectrum and the spectra at various pressures cross each other, i.e., they have the same S${}_{21}$ value at constant electron density. This frequency can also be seen in a similar simulation in [48]. In the current paper, this point was defined as the crossing frequency, $f_{\mathrm{cross}}$. The $f_{\mathrm{cross}}$ frequency is clearly seen even at high pressure where $f_{\mathrm{cutoff}}$ disappears. Hence, the proposed method of measuring $f_{\mathrm{cross}}$ can operate up to several tens of torr, providing a wider dynamic range than the $f_{\mathrm{cutoff}}$ method in terms of pressure. In fact, the $f_{\mathrm{cross}}$ is a direct function of electron density and sheath width and is independent of pressure. A detailed analysis of $f_{\mathrm{cross}}$ is discussed in the next section.

## 3. Experimental Demonstration and Circuit Model Analysis

### 3.1. Experimental Demonstration

**Figure 3:**
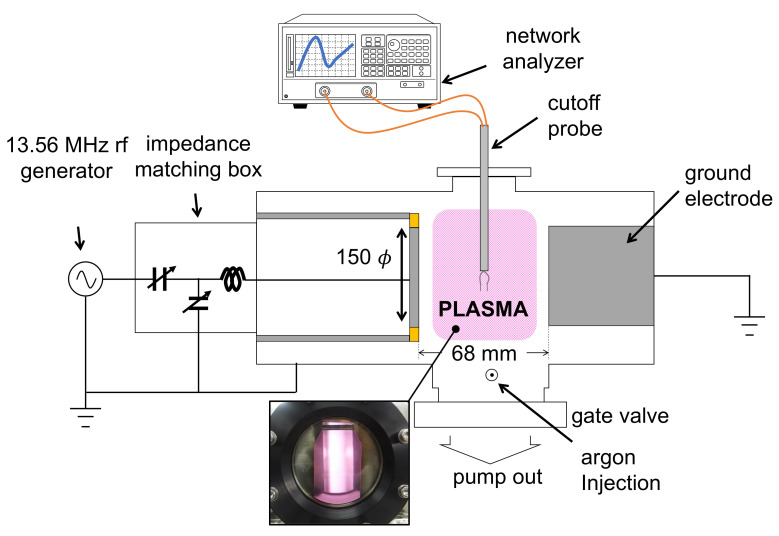
Schematic diagram of the experimental setup in a capacitively coupled plasma source.

Figure 3 shows a schematic diagram of the experimental setup. To demonstrate the crossing frequency method, a capacitively coupled plasma (CCP) was adopted. A 13.56 MHz power generator (RF5S, Advanced Energy Inc., Denver, CO, USA) was applied to a powered electrode with a diameter of 150 mm via an impedance matching box (PathFinder, Plasmart Inc., Daejeon, Korea) which maintains a load impedance of 50 Ω from the rf generator. The gap distance between the powered and grounded electrodes was 68 mm. The CP had a length of 5.0 mm, a tip distance of 5.0 mm, and a radius of 0.26 mm. The probe was inserted in the middle of the gap distance and connected with a vector network analyzer (S3601B, Saluki Technology Inc., Taipei, Taiwan) to measure the S${}_{21}$ spectrum. A rotary pump (GHP-800K, KODIVAC Ltd., Gyeong buk, Korea) and a turbomolecular pump (D-35614 Asslar, Pfeiffer Vacuum, Inc., Asslar, Germany) sustained a high-purity vacuum. The base pressure measured by a vacuum gauge (FullRange Gauge, Pfeiffer Vacuum, Inc, Asslar, Germany) was 5.4 × 10${}^{-7}$ Torr. Ar gas was injected into the chamber via a mass flow controller (MFC) (TN280, SMTEK CO., Ltd., Seongnam-si, Korea), and an MFC controller (GMC1200, ATOVAC Ltd., Yongin-si, Korea) maintained a constant gas flow rate of 50.0 sccm. The chamber pressure with Ar gas injection was measured by a precise vacuum gauge (Baratron 1 Torr, MKS Instruments Inc., Andover, MA, USA), and the pressure was controlled by changing the open and close ratio of a manual gate valve, as shown in Figure 3.

It is well known that in CCP discharge, the electron density depends on the pressure due to changes in the electron heating mechanism [54,55]. To demonstrate the proposed method, it is important to maintain the same electron density while the chamber pressure changes the same as in the simulation. To accomplish this, the rf power was slightly adjusted to preserve the same $f_{\mathrm{cutoff}}$ in the S${}_{21}$ spectrum. After $f_{\mathrm{cutoff}}$ vanished at high pressure (≃0.7 Torr), the rf power adjustment was no longer conducted. In [54,55], one can find that the electron density is nearly constant with a pressure above 1 Torr. Therefore, the adjustment approach in the current work was believed to be reasonable.

**Figure 4:**
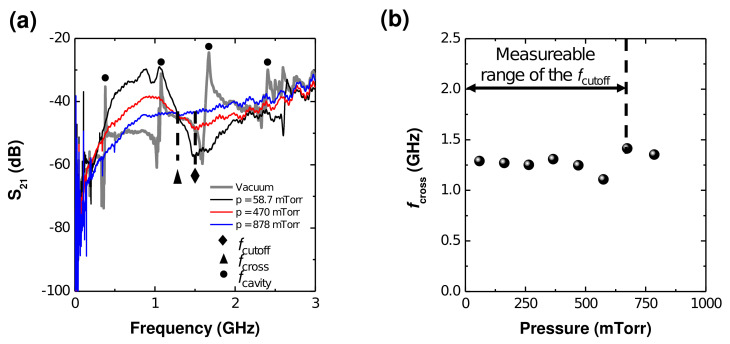
(**a**) Experimental S${}_{21}$ spectra at various pressures and vacuum with a cutoff frequency ($f_{\mathrm{cutoff}}$) of 1.5 GHz. (**b**) Measured $f_{\mathrm{cross}}$ at constant $f_{\mathrm{cutoff}}$ over pressure.

Figure 4a shows the experimental S${}_{21}$ spectra at vacuum and various pressure conditions. In contrast to the simulation results, the vacuum spectrum shows several resonance peaks that result from the chamber cavity effect [56], as the vacuum chamber is an electrical ground that forms a cavity structure. As shown in Figure 3, since the chamber dimension is long, the cavity resonance frequency forms in a relatively low frequency regime (several gigahertz). As for the plasma cases, there is a clear cutoff peak at low pressure that broadens with increasing pressure, showing the same trend as in the simulation results of Figure 2.

We note the clear $f_{\mathrm{cross}}$ in the experimental S${}_{21}$ spectra. Figure 4b plots $f_{\mathrm{cross}}$ over the chamber pressure and measurable range of $f_{\mathrm{cutoff}}$. As shown in that figure, the $f_{\mathrm{cutoff}}$ method is applicable below 700 mTorr, while $f_{\mathrm{cross}}$ shows a higher range. Since the vacuum gauge used in this experiment can measure pressure up to 1 Torr, higher pressure experiments were not conducted. Nevertheless, by combining the experimental and simulation results, the proposed method can reasonably be said to be applicable at several torr.

It should also be noted that the cavity peaks can distort the vacuum spectrum, which means that the vacuum spectrum does not cross $f_{\mathrm{cross}}$ even though the spectra from plasma do. While a full discussion of this point is beyond the scope of this paper, two solutions are briefly given as follows. The first is to remove the cavity resonance peaks through the time-gating method as in [57], which does not use continuous sinusoidal signals but pulse signals and removes the detection signals that include the cavity information in the time domain. The second approach is to choose an alternative reference signal, such as one of the pressure conditions shown in Figure 4a.

### 3.2. Circuit Model Analysis

**Figure 5:**
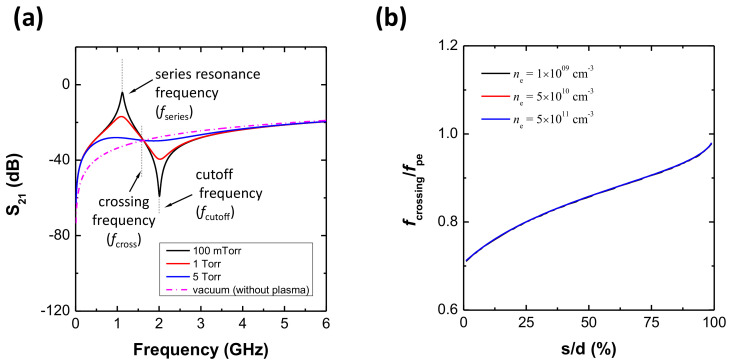
(**a**) S${}_{21}$ spectra at 100 mTorr, 1 Torr, 5 Torr, 10 Torr, and vacuum (without plasma) with an electron density of 5.0 × 10${}^{10}$ cm${}^{-3}$ and a fixed sheath width of 0.234 mm. (**b**) Normalized crossing frequency ($f_{\mathrm{crossing}}$/$f_{\mathrm{pe}}$) over the sheath width portion with antenna distance (*s*/*d*), where *s* and *d* are the sheath width and the antenna distance, respectively.

Until now, the operation of the $f_{\mathrm{cross}}$ method has been demonstrated by simulation and experiment. In this subsection, a circuit model analysis is provided to elucidate the meaning of the proposed method and to derive a relation between electron density and $f_{\mathrm{cross}}$. The circuit model is the same as in [48], and the same geometric parameters as in Section 2 are used in this model. Figure 5a shows S${}_{21}$ spectra at vacuum and various pressures, with the results representing that the circuit model well reproduces $f_{\mathrm{cross}}$ as well as the vanishing of $f_{\mathrm{cutoff}}$. Since the circuit model does not include electromagnetic effects such as cavity resonance, electron heating, etc., this analysis allows us to simply and clearly understand the $f_{\mathrm{cross}}$ method. Via the circuit model analysis, the $f_{\mathrm{cross}}$ method seems to have no limitation in terms of pressure. However, in practice, various causes such as cavity resonance, rf noise, instrument limitations, etc., distort the spectrum and give rise to the limitation of $f_{\mathrm{cross}}$ as shown in Section 3.1. Nevertheless, the model result implies that the $f_{\mathrm{cross}}$ method basically has a wider application window than that of the $f_{\mathrm{cutoff}}$ method.

Since the sheath width changes the plasma width, the $f_{\mathrm{cross}}$ was affected by the sheath width. To investigate this effect, Figure 5b shows a normalized crossing frequency, $f_{\mathrm{cross}}$/$f_{\mathrm{pe}}$, over the sheath width portion s/d at various electron densities, where $f_{\mathrm{pe}}$, *s* and *d* are the plasma oscillation frequency, sheath width, and antenna distance, respectively. The normalized frequency ranges from 0.7 to 1.0 over a sheath width portion from 1% to 99%, and interestingly, $f_{\mathrm{cross}}$/$f_{\mathrm{pe}}$ is independent of the electron density. Therefore, if *s* is provided, the electron density ($n_{e}$) can be obtained from $f_{\mathrm{pe}}$ ($f_{\mathrm{pe}}=8980\sqrt{n_{e}}$) by measuring $f_{\mathrm{cross}}$ based on Figure 5.

The sheath width around the CP can be estimated by measuring the series resonance frequency and the cutoff frequency in the S${}_{21}$ spectrum; this is well explained in [58]. However, as previously mentioned, $f_{\mathrm{cutoff}}$ disappears at high pressure, and thus estimating *s* becomes a challenge. To overcome this, one alternative way is to take the average as

(2) $$<g\left(x\right){>}_{\mathrm{avg}}=\frac{1}{99\;(\%)-1\;(\%)}\int_{1}^{99}g\left(x\right)dx≃0.85,$$

where *x* and g(x) are defined as s/d and $f_{\mathrm{cross}}/f_{\mathrm{pe}}$, respectively, with this equation having a standard deviation of 0.07. As a result, the averaged crossing frequency over sheath width, ${\bar{f}}_{\mathrm{cross}}$, is given by

(3) $${\bar{f}}_{\mathrm{cross}}=0.85\times f_{\mathrm{pe}}.$$

If other diagnostics to measure the sheath width are available, the best way is to measure the sheath width and derive the electron density from the result of Figure 5. However, if the sheath width is unknown, by using Equation (3), the electron density can be estimated with a theoretical discrepancy of about 10%.

## 4. Conclusions

This paper proposed a novel method to measure the crossing frequency $f_{\mathrm{cross}}$, which is applicable for high-pressure plasma diagnostics where the conventional $f_{\mathrm{cutoff}}$ method does not operate. The suggested method was demonstrated through both a CST simulation and an experiment with CCP discharge. Moreover, through the use of the circuit model, the relation between $f_{\mathrm{cross}}$ and $f_{\mathrm{pe}}$ was investigated. If the sheath width can be provided, the electron density can be estimated by measuring $f_{\mathrm{cross}}$ with the result from Figure 5. On the other hand, if the sheath width is unknown, by using Equation (3), the electron density can be estimated with a theoretical discrepancy of about 10%. In conclusion, the results show that the proposed method operates well in high pressure (several tens of torr) as well as low pressure.

## Data Availability

The data presented in this study are available on request from the corresponding author.
